# Influence of Prior Martensite on Bainite Transformation, Microstructures, and Mechanical Properties in Ultra-Fine Bainitic Steel

**DOI:** 10.3390/ma12030527

**Published:** 2019-02-12

**Authors:** Hui Guo, Xianying Feng, Aimin Zhao, Qiang Li, Jun Ma

**Affiliations:** 1 Facility Horticulture Laboratory of Universities in Shandong, Weifang University of Science and Technology, Weifang 262700, China; liqiangwky@126.com (Q.L.); majunwky@126.com (J.M.); 2 School of Mechanical Engineering, Shandong University, Jinan 250012, China; niuniubir@sina.cn; 3 Collaborative Innovation Center of Steel Technology, University of Science and Technology Beijing, Beijing 100083, China; zhaoaimin@ustb.edu.cn

**Keywords:** ultra-fine bainitic steel, prior martensite, incubation period, mechanical properties

## Abstract

A multiphase microstructure comprising of different volume fractions of prior martensite and ultra-fine bainite (bainitic ferrite and retained austenite) was obtained by quenching to certain temperatures, followed by isothermal bainitic transformation. The effect of the prior martensite transformation on the bainitic transformation behavior, microstructures, and mechanical properties were discussed. The results showed that the prior martensite accelerated the subsequent low-temperature bainite transformation, and the incubation period and completion time of the bainite reaction were significantly shortened. This phenomenon was attributed to the enhanced nucleation ratio caused by the introduced strain in austenite, due to the formation of prior martensite and a carbon partitioning between the prior martensite and retained austenite. Moreover, the prior martensite could influence the crystal growth direction of bainite ferrite, refine bainitic ferrite plates, and reduce the dimension of blocky retained austenite, all of which were responsible for improving the mechanical properties of the ultra-fine bainitic steel. When the content of the prior martensite reached 15%, the investigated steels had the best performance, which were 1800 MPa and 21% for the tensile strength and elongation, respectively. Unfortunately, the increased content of the prior martensite could lead to a worsening of the impact toughness.

## 1. Introduction

In the past decades, a kind of nanostructured high-carbon silicon-rich bainitic steel involving fine-scaled carbide-free bainitic ferrite plates and the uniform dispersion of carbon enriched austenite was developed by Caballero and Bhadeshia [1]. These bainite steels exhibit a remarkable combination of ultrahigh strength and excellent toughness by isothermal treatment at a low temperature, which has incredible potential in the transport, construction, and offshore industries, as well as in defense applications [2,3]. The prominent mechanical properties have aroused widespread concern from institutes to industries.

Nevertheless, the isothermal bainite transformation cycle is very time consuming, taking up to even a month, and the tediously lengthy heat treatment limits its industrial application. Therefore, shortening the holding time, without sacrificing the benefit of ultra-fine bainitic steels, has become a hot issue in this field. Recently, many researchers have been striving to explore the transformation acceleration mechanism, which contains composition optimization [4,5,6,7], grain refinement [8,9], heating treating optimization [10,11,12], applying stress to austenite [13,14,15,16], and introduced a partial phase [17,18,19,20]. Thereafter, the new process of producing a prior phase before the bainite transformation aroused attention. Kawata [17] claimed that the interfacial energy introduced by the prior martensite (hereinafter referred to as PM) played a major role in the acceleration of the bainite transformation. Gong et al. [21] reported that the prior martensite transformation could accelerate the subsequent nanobainite transformation. Toji [22] also showed that the acceleration effect of PM existed in both Si-containing and Si-free steels. Although, the effect of PM on the microstructure evolution and bainite kinetics has been characterized. Several investigations have been conducted to evaluate the effect of the PM on the microstructure evolution and bainite kinetics, yet the mechanism behind the acceleration of the bainite transformation and the mechanical properties remain unclear.

Therefore, a new composition of high-carbon low-alloy ultra-fine bainitic steel was designed in this study, and a given fraction of martensite was introduced by quenching to a certain temperature between Ms and Mf (the martensite transformation start and finish temperature, respectively), followed by up-heating to the isothermal bainite transformation temperature. Furthermore, the effect of the prior martensite on the ultra-fine bainite transformation incubation period, transformation kinetics, and microstructures, as well as the mechanical properties were quantitatively analyzed.

## 2. Materials and Methods 

**Figure 1:**
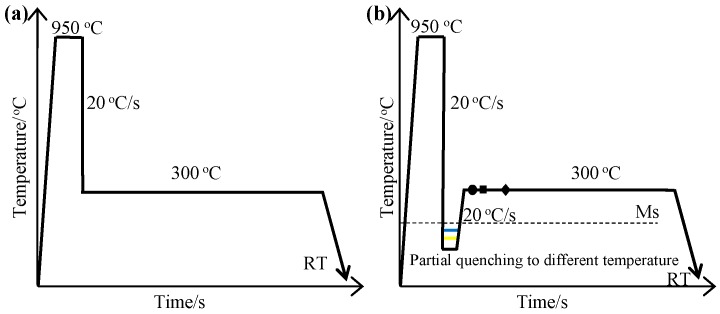
Schematic diagram of different heat treatments. (**a**) DIT process: direct isothermal bainite transformation. (**b**) QBT process: partial quenching to different temperature and subsequent isothermal bainite transformation; the circle, square, and diamond stand for the different isothermal holding time, respectively.

The chemical composition of the investigated steel was Fe–0.7C–2.47Si–1.46Mn–0.87Al–0.02Nb (wt.%). Si and Al were added to inhibit carbide precipitation during the austempering process. The alloy was prepared by 50 kg vacuum melting and was casted into ingots. The ingots were forged into square bars with dimensions of 150 mm × 70 mm × 30 mm, and then homogenized at 1200 °C for 36 h in a vacuum furnace. Cylindrical samples (Φ 4 mm × 10 mm) were heat treated with a DIL805A dilatometer (BÄHR-Thermoanalyse GmbH, Hüllhorst, Germany), in which heating was performed by an induction coil under the vacuum of the order of 10^−4^ bar, and by cooling using a continuous flow of helium gas, in order to measure the Ms temperature and to quantitatively evaluate the bainite transformation time and phase transformation temperature. The tensile specimens were thermally treated with a 70 mm × 25 mm × 15 mm salt bath furnace. Two heat treatments were carried out as shown in Figure 1, as follows: (a) direct isothermal bainite transformation (DIT), and (b) partial quenching to different temperatures, and subsequent isothermal bainite transformation (QBT). In the DIT process, the samples were cooled from 950 directly to 300 °C for the isothermal bainite transformation. In the QBT process, the samples were initially austenitized at 950 °C for 10 min, then quenched to several temperatures at a cooling rate of approximately 20 °C/s, which was lower than the Ms temperature (190 °C) by over 5, 7, 12, 18, and 30 °C, in order to achieve different martensite percentages, and were then kept for 10 s to reach a homogeneous temperature, subsequently heated up to 300 °C for isothermal holding, and were finally cooled to room temperature. Correspondingly, the samples were referred to as QBT-5, QBT-7, QBT-12, QBT-18, and QBT-30, respectively. The austempering time at 300 °C for DIT and QBT process were both about 1, 5 and 10 min and 4 h. According to the Koistinen–Marburger (KM) equation [23], which describes the volume fraction of the transformed martensite as a function of the primary quenching temperature, the volume fraction of the prior martensite was approximately 5%, 10%, 15%, 20%, and 30%, respectively.

The microstructural characterizations were analyzed using a ZEISS AX10 light microscope (LM, Carl Zeiss, Thornwood, NY, USA), ZEISS ULTRA 55-type field emission scanning electron microscope (SEM, Carl Zeiss, Thornwood, NY, USA), and Tecnai G2 F30 S-TWIN transmission electron microscope (TEM, FEI Company, Hillsboro, OR, USA). The metallographic samples for the LM and SEM were ground, mechanically polished, and etched with a 2% Nital solution. Image Pro Plus software (Version 6.0, Media Cybernetics, Rockville, MD, USA) was adopted to quantitatively evaluate the microstructure. The thin foils used for the TEM were sliced into 0.3 mm from the cylindrical samples, ground down to 50 μm, and then electropolished at 50 V by a twin jet unit using an electrolyte solution of 5% perchloric acid. Electron back-scattering diffraction (EBSD) was employed in FE-SEM with 20 kV and a step size of 0.05 μm. Data acquisition and analysis were performed by means of the CHANNEL HKL 5 system (Oxford Instruments, Bicester, UK). The quantitative analysis of the retained austenite was determined by D8 ADVANCE Twin X-ray diffraction (XRD, Bruker, Hamburg, Germany) using Cu-Kα radiation, with a voltage of 40 kV and a current of 50 mA. The XRD data were collected from a range of 47°–93° with a step of 1°. The samples for the EBSD and XRD were ground and electrolytically polished with 20% perchloric acid and 80% ethanol at 15 V for 20 s.

The tensile tests were performed on a MTS 810 tensile testing machine (MTS Corporation, Eden Prairie, MN, USA) at room temperature, with a gauge diameter of 5 mm and gauge length 25 mm. The cross-head speed was 0.1 mm/min, corresponding to an engineering strain rate of 6.66 × 10^−5^ s^−1^. The engineering stress–strain curves that were measured during the uniaxial tensile tests were converted into the true stress–strain curves, and the instantaneous work hardening exponent was deduced from the stress–strain curve [24]. The yield stress (YS) was determined by the 0.2% offset method. Charpy impact tests were conducted on 10 mm × 10 mm × 55 mm notched samples at ambient temperature. The hardness measurement of the samples was conducted using a Rockwell microhardness tester machine with a load of 10 kgf, and at least five readings per condition were taken and the average values were reported in each case.

## 3. Results

### 3.1. Bainite Transformation Kinetic

**Figure 2:**
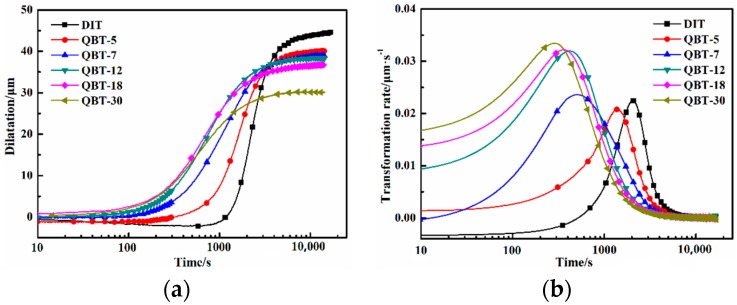
Comparison of bainite transformation kinetics between DIT and QBT samples during isothermal treatment at 300 °C. (**a**) Dilatation–time curves; (**b**) transformation rate curves.

Figure 2 shows the relative change in length with respect to the temperature of the DIT and QBT samples during isothermal treatment at 300 °C. Generally speaking, the time at which a 1% and 98% volume fraction of bainite is generated is considered as the start and completion time of the isothermal bainite transformation. The relative results of the various samples are listed in Table 1. From the dilatation–time curves (Figure 2a), the time required for the initiation of the bainite transformation decreases with the increasing of the prior martensite fraction, namely, the incubation period reduces. Figure 2b displays the corresponding transformation rate of Figure 2a, which is defined as the dilatation at each time, divided by the maximum dilatation. The transformation rate rapidly increases with the increase in the prior martensite fraction, and the time corresponding to the peak rate decreases. Therefore, the bainite transformation is obviously accelerated by the introduced prior martensite. This phenomenon may attribute to the condition change for nucleation and growth of the bainite isothermal transformation and the decreased required energy for heterogeneous nucleation, caused by the increased dislocation density and stress fields, due to the quenched martensite phase transformation expansion [22]. With the increasing prior martensite fraction, the nucleation sites and nucleation rate notably increase, leading to a shortened incubation period and enhanced transformation rate.

It is widely acknowledged that the incomplete transformation phenomenon is the main characteristic of the isothermal bainite reaction, and the maximum attainable volume fraction of bainitic ferrite is subjected to the carbon concentration of the retained austenite, without exceeding the *T*_0_ curve (i.e., *X_T_*_0_) [25]. The curve stands for the locus of points where ferrite and austenite of the same chemical composition have equal free energy. At this stage, the carbon-enriched retained austenite would not further transformation into bainitic ferrite. During the austempering reaction, the excess carbon from the supersaturated prior martensite will partition into the adjacent untransformed austenite [22], which can increase the stability of the retained austenite. Therefore, corresponding to the DIT sample, the QBT process reduces the time that the carbon concentration of the retained austenite reaches the *X_T_*_0_, and the completion time of the isothermal bainite transformation is shortened.

### 3.2. Microstructural Characteristics

**Figure 3:**
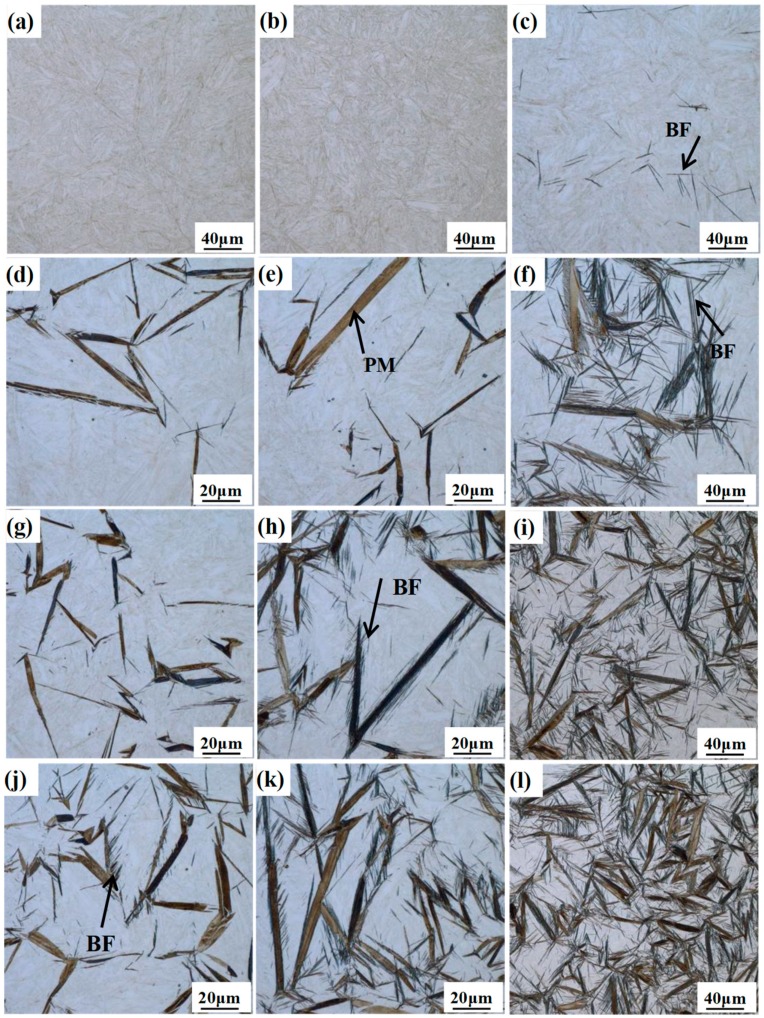
ZEISS AX10 light microscope (LM) micrographs of different samples transformed at 300 °C for different times. (**a**–**c**) are the DIT sample austempering for 1, 5, and 10 min, respectively; (**d**–**f**) are the QBT-5 sample austempering for 1, 5, and 10 min, respectively; (**g**–**i**) are the QBT-7 sample austempering for 1, 5, and 10 min, respectively; (**j**–**l**) are the QBT-12 sample austempering for 1, 5, and 10 min, respectively. BF refers to bainitic ferrite, and PM refers to prior martensite.

Figure 3 presents the LM micrographs of the different samples for isothermal treatment at 300 °C for the different times. With the increase of the isothermal holding time, the volume fraction of the bainitic ferrite gradually increases, but the dimension of the bainitic ferrite plates exhibits an obvious difference. In the case of the DIT treatment for 1 and 5 min, only thinly-etched fresh martensite can be observed (Figure 3a,b), which generates upon final quenching to room temperature. A slight nucleation of bainitic ferrite with a black contrast is detected after 10 min (Figure 3c). However, the transformation proceeds much faster in the QBT treatment, and the relatively large plates with a black contrast refer to the needle-like prior martensite and the tiny gray ones to the bainite. Comparing Figure 3d,g,j, only the QBT-12 (Figure 3j) sample involves a small piece of plate-shaped bainitic ferrite near the prior martensite plates. When the isothermal time further increases to 5 min, the QBT-7 (Figure 3h) sample appears to be a bainite microstructure, however, the QBT-5 (Figure 3e) sample only exists in prior and fresh martensite. The observation also confirmed that the bainite transformation could be significantly accelerated by the formed prior martensite. It can be perceived that bainite laths quickly nucleate at the martensite–austenite interfaces, and the increased volume fraction of the prior martensite provides a favorable prerequisite for bainite nucleation, resulting in improving the bainite transformation rate and a shortening incubation period. 

**Figure 4:**
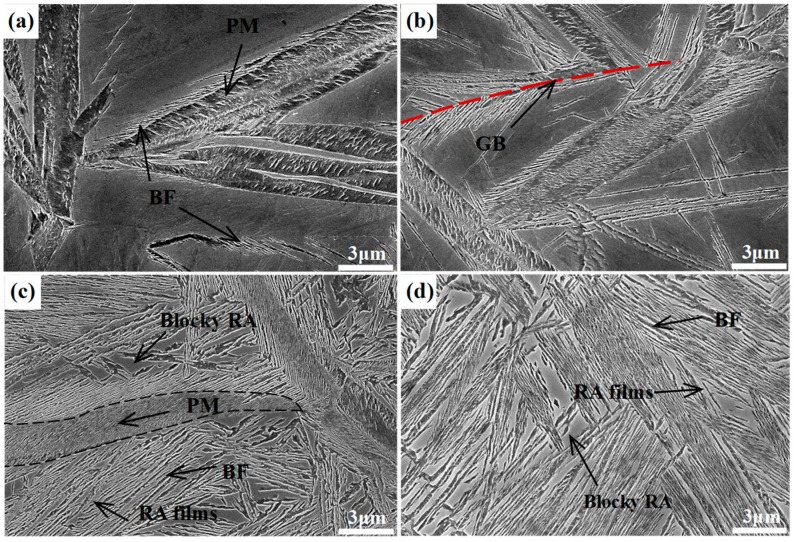
SEM micrographs of different samples after isothermal treatment at 300 °C for different times. (**a**–**c**) are the QBT-12 sample austempering for 1 min, 5 min, and 4 h, respectively; (**d**) is the DIT sample austempering for 4 h. RA films refer to film-like retained austenite, Blocky RA to blocky retained austenite, GB to grain boundary, and PM to prior martensite.

**Figure 5:**
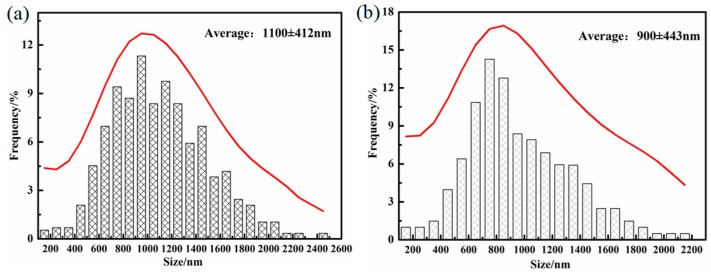
Size distribution of the blocky retained austenite in different samples after isothermally transformed at 300 °C for 4 h. (**a**) DIT sample; (**b**) QBT-12 sample.

Figure 4 shows the SEM micrographs of the different samples transformed at 300 °C for different times, in which the lower relief phase corresponds to the bainitic ferrite plates, and the higher relief phase to the retained austenite. The austenite presents in two main morphologies, the exemplary island type morphology of a micro-/submicron-scale blocky retained austenite, which locates between the sheaves of bainitic ferrite, and the nanoscale film-like retained austenite, which is reserved between the bainitic ferrite plates. In addition, the QBT sample comprises a little lenticular martensite with a nonuniformly color inside the lath, which exhibits the metallurgical characteristic of tempered martensite. As shown in Figure 4a, the QBT-12 sample has already existed bainitic ferrite near the prior martensite plates after austempering for 1 min, and the bainite plates are initially formed adjacent to the prior martensite and grew up to austenite, which has also been confirmed by the in-situ observation [26]. When the isothermal time increases to 5 min, the bainite just nucleates at the prior austenite grain boundaries, and sheaves grow towards the grain interior. Figure 4c shows the SEM micrographs of the QBT-12 sample austempering for 4 h, and it can be seen that the thickness of the bainitic sheaves adjacent to the prior martensite plates become refined. The explanations therefore may predominantly consist of the following three main constituents: (a) the formation of martensite increases the density of the potential nucleation sites for bainitic ferrite, leading to the refinement of bainite microstructure; (b) the prior martensite divides the austenite grain and reduces the scale of the untransformed austenite; (c) the prior martensite induces a stress–strain field, which can provide extra activation energy for nucleation and constrain the free movement of bainite–austenite, which is responsible for the refinement of bainitic ferrite laths [21]. All of these effects are likely to contribute towards developing an ultrafine microstructure, but it is difficult to quantitatively distinguish which factor is dominant. Moreover, the QBT process can effectively reduce the size and volume fraction of the blocky retained austenite in relation to the DIT, and the specific statistical results are presented in Figure 5. Both sizes of retained austenite are subject to normal distribution in the DIT and QBT-12 samples, and the average size of the blocky retained austenite would be reduced from 1100 to 900 nm.

**Figure 6:**
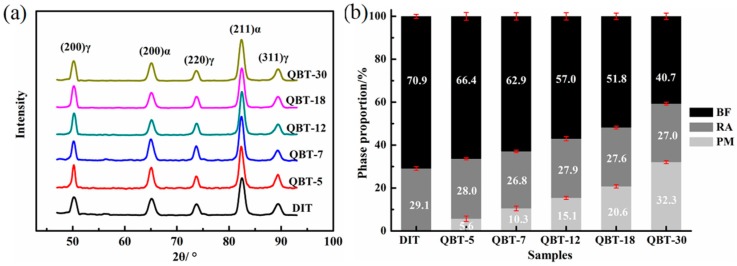
XRD patterns (**a**) and phase proportion (**b**) of different samples after isothermal treatment at 300 °C for 4 h.

The XRD patterns and phase proportion of the different samples after isothermal treatment at 300 °C for 4 h are shown in Figure 6. It is clear that only the BCC phase and FCC phase diffraction peaks can be observed in Figure 6a, namely, no carbide precipitation is formed during the bainite transformation. Corresponding to the DIT sample, the volume percentage of the bainitic ferrite in the QBT samples reduces, and with the increase in the prior martensite fraction, there is a significant decrease in the formation of bainitic ferrite, however, the content of the retained austenite remains almost the same. This can be explained by the fact that the prior martensite consumes partial supercooled austenite, and the increased bainitic ferrite plates adjacent to the prior martensite plates increase the probability of the impingement of bainite laths upon growth, resulting in a reduction in the maximum attainable volume fraction of bainite.

**Figure 7:**
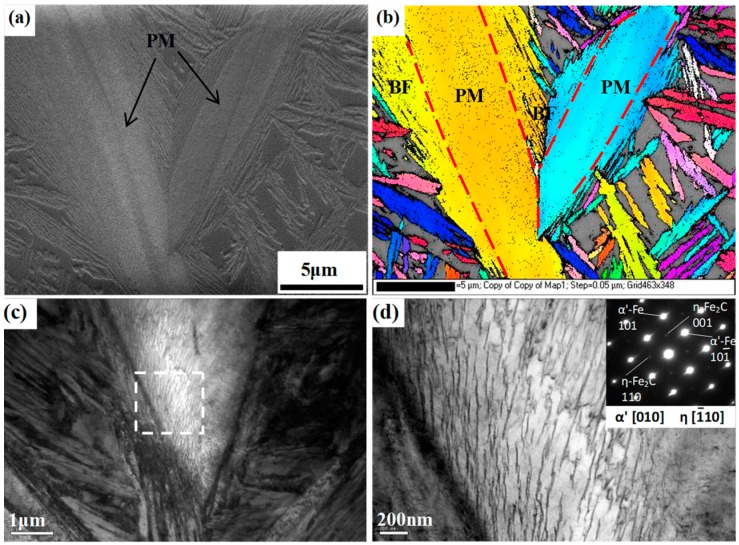
Microstructural characteristics of the QBT-12 sample. (**a**) SEM and the corresponding electron back-scattering diffraction (EBSD) micrograph (**b**); (**c**) TEM micrograph and (**d**) the high magnification observation of the framed area in (**c**).

Figure 7 shows the EBSD and TEM micrographs of the martensite plates and the surrounding bainite sheaves in the QBT sample to further reveal the fine details of the microstructure, in which the color refers to the crystallographic orientation perpendicular to the observation plane, and various colors stand for the different crystallographic orientations. It is interesting to note that the martensite plate and the adjacent group of bainitic ferrite share a common crystallographic orientation, and the orientation of bainite seems to be regulated by the adjacent martensite; a similar orientation regulation effect by prior martensite has also been reported in the literature [21]. Meanwhile, according to the results of Miyamoto [27], the thickness of the surrounding bainite sheave and plastic deformation region of the surrounding martensite have a relatively high level of consistency, which indicates that the introduction of prior martensite can bring about the deformation of the surrounding austenite and deflect its orientation, which, as a result, exerts an effect on the growth direction of the critical bainite laths. Based on the available literature and the observed results, it is inferred that the strain caused by the prior martensite that provided the preferential nucleation sites is a more dominate factor than the interface between the martensite and austenite for the acceleration effect of the ultra-fine bainitic steel. The TEM image in Figure 7c confirms that the bainitic ferrite plates nucleate on the prior martensite lath boundaries and have an average thickness of 50 nm, and the refined bainitic ferrite are attributed to the increased nucleation sites by prior martensite transformation. Furthermore, Figure 7d demonstrates that the intermittent chain arrangement transition type of the precipitated η-carbide inside the PM laths can be formed.

**Figure 8:**
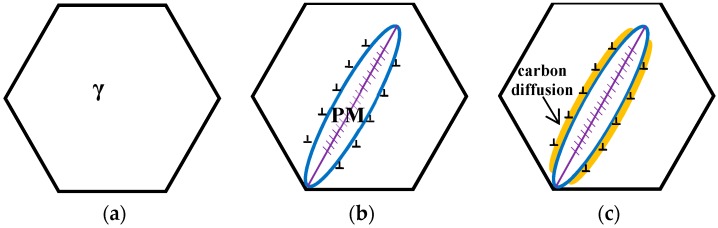

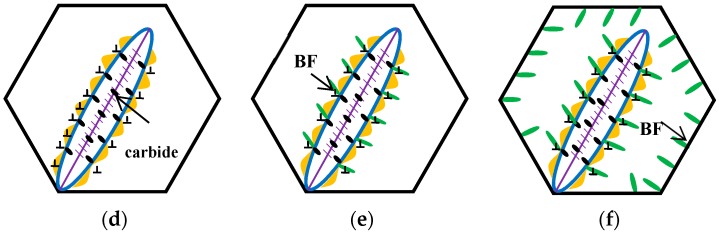
Schematics diagrams of QBT process.

The schematic diagrams of the prior martensite transformation are shown in Figure 8. Firstly, a certain quantity of martensite with twins along the midrib, can be generated after complete austenitizing (Figure 8a) and partial quenching to the temperature below the martensite point; meanwhile, the prior austenite grains are divided into multiple regions by prior martensite (Figure 8b). The martensite transformation can bring out the volume expansion and the increase of the dislocation density of the surrounding austenite (Figure 8c). Afterwards, there is a partitioning phenomenon between the prior martensite and retained austenite, thus the carbon atoms in the prior martensite are rejected into the surrounding austenite within the isothermal stage under the Ms temperature, leading to the carbon concentration of the austenite increase near the interface. When heated up to 300 °C, the carbon concentration of austenite, at least the local carbon concentration, is already higher than the value of *T*_0_ line. At the same time, the carbides can be formed inside the prior martensite by carbon migrating (Figure 8d), resulting in the region close to the interface, which is away from carbides, having a high carbon concentration. Meanwhile, the region adjacent to the carbides is prone to exhibit a low carbon concentration, because carbon atoms have been consumed inside PM. Under this circumstance, the bainite laths nucleates at the C-depleted region, close to the precipitated carbides (Figure 8e). With increasing the holding time, some of the bainite connect to the boundary of the martensite and austenite, and others grow in the direction of the grains interior (Figure 8f).

### 3.3. Mechanical Properties

The existing prior martensite has a profound effect on the isothermal bainite transformation kinetics and microstructure, and, therefore, would bring about the change in the mechanical properties. The tensile and impact tests are carried to evaluate the difference of the mechanical behavior affected by the prior martensite. 

**Figure 9:**
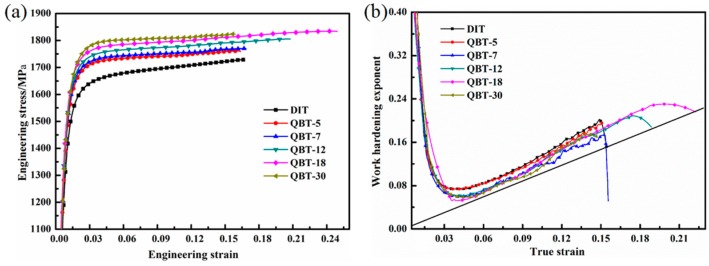
(**a**) Engineering stress–strain curves and (**b**) corresponding instantaneous work hardening exponent as a function of the true stain curves of the investigated steel after isothermal treatment at 300 °C for 4 h.

Figure 9a shows the engineering stress–strain curves of ultra-fine bainitic steel obtained by different processes, and the corresponding mechanical properties are summarized in Table 2. It can be seen that both the strength and elongation are enhanced after the QBT process, and all curves are characterized by the presence of the continuous yielding behavior within the whole tensile strain range, as well as slight necking before failure. The yield strength and ultimate tensile strength gradually improve as the prior martensite content raises from 5% to 30%; however, the total elongation increases initially and then declines afterwards, and there is a slight decline when the volume fraction of prior martensite reaches to 30%. The strengthening mechanisms of the ultra-fine bainitic steel mainly give the credit to the scale of the bainite plates, level of crystal defects, and carbon concentration of solid solution. Two possible aspects could be employed to explain the improvement of strength. Firstly, the existing martensite can impede the dislocation glide and the propagation located in the bainitic ferrite, and hence, in a microscopic view strengthens the bainitic ferrite [28]. Moreover, the prior martensite can effectively improve the nucleation rate of the bainitic ferrite, resulting in the refinement of bainite plates, leading to a reduction in the mean free path for dislocation. As the extraordinarily slender plates and substructure unit are critical for obtaining good strength, there is no apparent difference on the strength of the different processes.

The ductility of the ultra-fine bainitic steel primarily arises from the volume fraction, morphology, carbon concentration, and distribution state of the retained austenite. As a result, the existing prior martensite can reduce the scale and content of the blocky untransformed austenite, leading to a uniform plastic deformation during the tensile process, therefore bringing about additional ductility, such as the QBT-12 and QBT-18 samples. Lan and Avishan also suggested that it would be favorable to replace blocky RA with filmy RA as much as possible, as it is effective in ductility [29]. Unsatisfactorily, when the prior martensite increases to 30% (i.e., QBT-30), the total elongation trends to drop and is lower than the DIT sample. This phenomenon can be explained that the carbon atom in the prior martensite partitioning towards the adjacent untransformed austenite, which has been confirmed by atom probe tomography [22], and can result in the change of carbon concentration in untransformed austenite, which influences the stability of the partial retained austenite. The majority of the austenite remained untransformed in the necked region, and hence cannot fully exploit its beneficial effect on the ductility. 

Figure 9b shows the curves of the instantaneous work hardening exponent (n) versus the true strain. The straight line corresponds to the instability criterion *n* = ε_u_, in which ε_u_ stands for the true strain at the beginning of necking. Both samples have some relevant features in common, a high increase of n during the first stage of plastic deformation, followed by a drastic drop that ends at low levels of plastic deformation. It is probably because the retained austenite in the sample is less stable at the strain-induced martensite transformation at the small plastic strain, subsequently, the transformation-induced plasticity (TRIP) effect exhibits a better strain-hardening at the latter stage of deformation, consequently postponing the onset of the necking up to a high strain region. Compared with the DIT sample, the QBT-12 and QBT-18 samples exhibit a remarkable continuous increase in the work hardening exponent after a sharp decrease at a low strain regime. Much scientific research shows that the large blocky type retained austenite has a lower carbon and more inhomogeneous carbon distribution, and in turn a low mechanical stability than that in the small one, therefore, the strain induced transformation occurs at the early stage of deformation, which contributes much less to the TRIP effect. Meanwhile, the overly coarse hard phase (martensite) from the coarse retained austenite accelerates the initiation and propagation of the microcracks, resulting in losing the protection against the necking instability. However, the small-sized blocky and film-like retained austenite with a higher stability, which can transform in a progressive manner into martensite upon deformation, leading to the enhanced ductility and an increase in the work hardening rate that delays necking phenomenon. 

**Figure 10:**
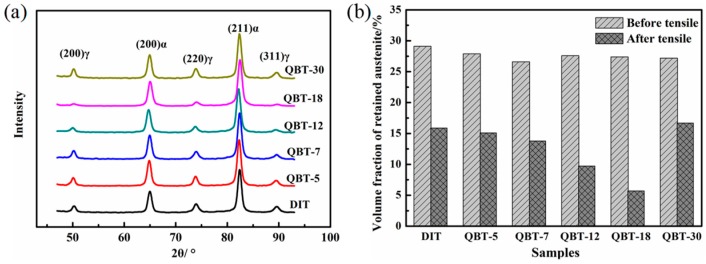
(**a**) XRD spectra and (**b**) volume fraction of retained austenite after tensile tests.

In order to examine the change in the proportion of retained austenite after tensile tests, the XRD results are shown in Figure 10. The decrement of the retained austenite during the tensile is measured to be 13.2%, 12.8%, 12.8%, 17.9%, 21.7%, and 10.5% for the DIT, QBT-5, QBT-7, QBT-12, QBT-18, and QBT-30 samples, respectively, and there is still a certain amount of untransformed retained austenite close to the fracture surface. Therefore, the change of quantity of the retained austenite of the QBT-12 and QBT-18 samples are the most obvious, which leads to excellent plastic deformation behavior.

**Figure 11:**
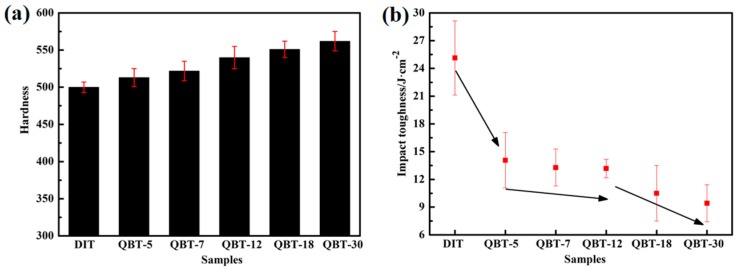
Mechanical properties of investigated steel. (**a**) hardness; (**b**) impact toughness.

The hardness of the different samples is given in Table 2 and Figure 11a. The data present that the hardness of the QBT samples is superior to the DIT sample, and with the increase of prior martensite fraction, the hardness gradually enhances, therefore the hardness of the QBT-30 sample is found to be more than 10% when compared to the DIT sample. This fact is consistent with the higher UTS obtained from the engineering stress–strain curve. The enhanced average hardness is attributed primarily to the increased volume fraction of the harder microstructure of martensite and the refined microstructure.

By contrast, the impact toughness of the ultra-fine bainitic steel exhibits the opposite trend; the increased volume fraction of the prior martensite can lead to the impact toughness worsening, as shown in Figure 11b. It is well known that the impact absorbed energy mainly consists of crack initiation energy and crack propagation energy. Compared to the QBT samples, the DIT sample with a larger proportion of metastable blocky retained austenite will transform to martensite during the crack initiation region, which can absorb more energy and provide crack blunting and crack closure because of the compression caused by dilatation; as a consequence, it benefits to increase the crack initiation energy. However, a great deal of martensite provides more nucleation sites for the microvoid, which greatly contributes to the rapid development of the crack and decreasing the crack propagation energy. Whereas, the QBT process can improve the stability of retained austenite, leading to the martensite transformation consuming more energy, which could decrease the generated martensite propitiation of the crack initiation stage, delay the void nucleation, and reduce the net energy available for the crack growth because of the energy dissipation. It appears that the impact energy of the QBT samples are significantly lower than the DIT sample, which means that the introduced prior martensite exerts an influence on the impact properties, even though after a long time of tempering, it still deteriorates the impact properties. This is not agreement with the results reported by Garbarz [30], which mainly relate to the steel composition and transformation time, therefore, the paper adopts a low-carbon system, which has a high ductile compared to the high carbon martensite. As can be seen from Figure 11b, when the prior martensite percentage increases from 6% to 15%, the impact toughness of the investigated steel keeps almost unchanged, which may be the result of the concurrent effects of the refined microstructure and decreased blocky retained austenite by martensite transformation. The toughness of the ultra-fine bainitic steel is usually governed by the volume fraction of the ductile phase retained austenite, and its stability towards stress or strain induced transformation to martensite. Microstructure refinement is beneficial to reduce stress centralizing and decrease the probability of crack initiation; simultaneously, the increased interface can effectively resist crack propagation. Nevertheless, blocky retained austenite with the lower thermal and mechanical stability, especially the central parts, is prone to form martensite upon the initial stage of deformation, which is detrimental to the impact properties. The film-like retained austenite can effectively block the crack propagation channel and deplete the crack propagation energy, and accordingly, play a role in blunting the crack tip, slowing down the crack growth rate, relieving the stress concentration, and closing the crack by absorbing relatively more energy. It is noteworthy that when the prior martensite percentage exceeds 15%, the impact properties can be further decreased.

**Figure 12:**
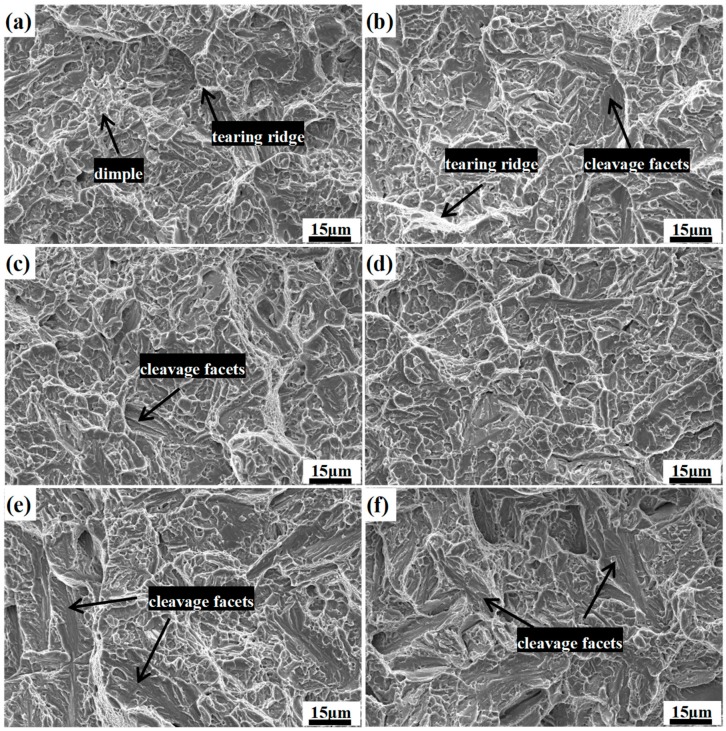
Microfracture morphology of fracture surface in different impact samples. (**a**) DIT; (**b**) QBT-5; (**c**) QBT-7; (**d**) QBT-12; (**e**) QBT-18; (**f**) QBT-30.

Figure 12 shows the typical SEM microfracture morphology from the center region of the fractures surface in the different impact samples. It is quite notable that the appearance of the fractured surface involves cleavage facets, tear ridges, and dimples in all of the samples, showing a signature of a combination of ductile fracture and brittle fracture. As shown in Figure 12a, the fractography of the DIT sample is characterized by a great many curved features connected together with the aspect of the ductile fracture. However, with an increase in the volume fraction of the prior martensite, the features of the cleavage fractures gradually increase, while the amount of dimples and tear ridges slightly decrease, which shows a quasi-cleavage type of fracture; in particular, the fractography of QBT-30 sample, which shows an angular surface with multiple cleavage planes due to a low toughness of about 10 J.

**Figure 13:**
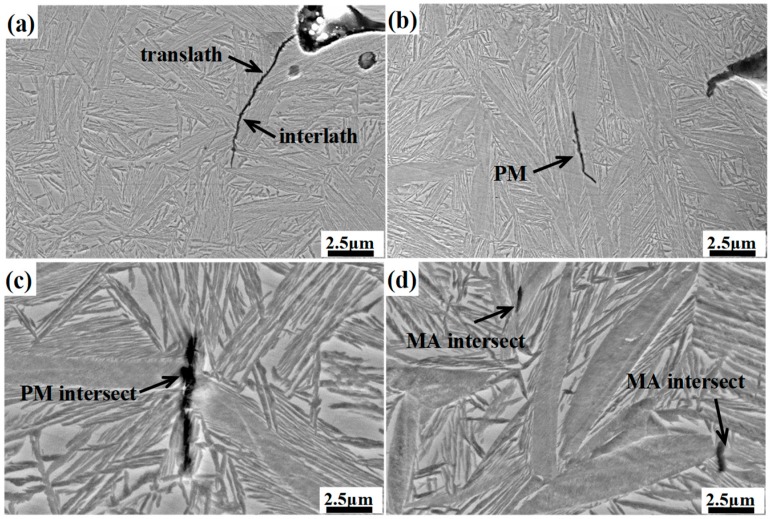
Micrograph of longitudinal section of different impact samples. (**a**) DIT sample; (**b**–**d**) QBT sample.

Figure 13 shows the micrograph of the longitudinal section of the different impact samples. For the DIT sample, the cracks mainly expand along the bainitic sheaves boundaries and film-like retained austenite, and a small amount of cracks cross through the bainitic ferrite and film-like retained austenite. The former is called an interlath crack, and the latter is called translath crack [31]. In the case of the samples with prior martensite, the cracks mainly spread along the prior martensite intersection or the junction of the martensite and the retained austenite. The prior martensite is considered to be a hard–brittle phase, which may exist as residual stress at the interface, and the stress concentration between the martensite and retained austenite can be easily generated because of the greater strength mismatch between them; therefore, the crack will be initially nucleated and extended at those sites, resulting in the degradation of the impact properties.

## 4. Conclusions

The bainite transformation kinetics and mechanical properties of ultra-fine bainitic steel by a novel QBT process have been evaluated. It can be concluded that the prior martensite had a significant effect on the isothermal bainite transformation. With increasing the prior martensite fraction, the nucleation sites and nucleation ratio of the bainitic ferrite near the martensite plates increased, while the incubation period and completion time of the bainitic transformation were significantly shortened. The prior martensite plates influenced the crystal growth of the adjacent bainite, which resulted in the bainitic lathes that formed adjacent to the prior martensite plate sharing an almost identical orientation. The prior martensite transformation could effectively reduce the scale of the blocky retained austenite; refine the bainitic microstructure; and improve the hardness, strength, and ductility of the investigated steels, but slightly deteriorate the impact properties. In summary, the QBT process was an effective way to develop ultra-fine bainitic steel, and the optimum combination of strength, elongation, and impact toughness could be achieved by controlling the prior martensite content to 15%.

## Figures and Tables

**Table 1:** Bainite transformation time and maximum transformation rate.

Samples	*T*_s_/s	*T*_f_/s	ε_max_/μm·s^−1^
QBT-30	2	5046	0.033
QBT-18	5	5047	0.032
QBT-12	38	5500	0.031
QBT-7	89	6330	0.023
QBT-5	392	6760	0.021
DIT	1215	8320	0.022

Where *T*_s_, *T*_f_, and ε_max_ stand for the start time, completion time, and maximum rate of the bainite transformation, respectively. DIT: direct isothermal bainite transformation; QBT: partial quenching to different temperatures, and subsequent isothermal bainite transformation.

**Table 2:** Summary of mechanical properties of DIT and QBT samples obtained from various tests performed at room temperature.

Samples	YS	UTS	TE	PSE	IE	Hardness
	MPa	MPa	%	GPa·%	J	HV(10)
QBT-30	1416 ± 3	1822 ± 3	15.9 ± 1.3	29.0	9.42 ± 2	562 ± 13
QBT-18	1412 ± 5	1834 ± 7	25.0 ± 1.8	45.8	10.5 ± 3	551 ± 11
QBT-12	1400 ± 3	1805 ± 8	20.8 ± 0.9	37.5	13.17 ± 1	540 ± 15
QBT-7	1354 ± 6	1770 ± 4	16.8 ± 1.6	29.7	13.28 ± 2	522 ± 13
QBT-5	1340 ± 5	1761 ± 3	16.4 ± 0.5	28.9	14.07 ± 3	513 ± 12
DIT	1284 ± 9	1729 ± 2	16.7 ± 1.2	28.8	25.13 ± 4	500 ± 7

Where YS, UTS, TE, PSE, and IE stand for yield strength, ultimate tensile strength, total elongation, the product of ultimate tensile strength and total elongation, and Charpy impact energy, respectively.
